# X-ray Determination of Compressive Residual Stresses in Spring Steel Generated by High-Speed Water Quenching

**DOI:** 10.3390/ma12071154

**Published:** 2019-04-09

**Authors:** Diego E. Lozano, George E. Totten, Yaneth Bedolla-Gil, Martha Guerrero-Mata, Marcel Carpio, Gabriela M. Martinez-Cazares

**Affiliations:** 1 Departamento de Ingeniería, Universidad de Monterrey, Av. Morones Prieto 4500, San Pedro Garza García 66238, Mexico; diego.lozanod@udem.edu (D.E.L.); yaneth.bedolla@udem.edu (Y.B.-G.); 2 Department of Mechanical and Materials Engineering, Portland State University, Portland, OR 97201, USA; getotten@gmail.com; 3 Facultad de Ingeniería Mecánica y Eléctrica, Universidad Autónoma de Nuevo León, Pedro de Alba s/n, San Nicolás de los Garza 66455, Mexico; mpgmus@yahoo.com.mx; 4 Departamento de Ciencia de los Materiales e Ingeniería Metalúrgica, EBBE, Universitat Politecnica de Catalunya, Av. Eduard Maristany 16, 08019 Barcelona, Spain; marcel.francisco.carpio@upc.edu

**Keywords:** residual stresses, X-ray, quenching, steel

## Abstract

Automotive components manufacturers use the 5160 steel in leaf and coil springs. The industrial heat treatment process consists in austenitizing followed by the oil quenching and tempering process. Typically, compressive residual stresses are induced by shot peening on the surface of automotive springs to bestow compressive residual stresses that improve the fatigue resistance and increase the service life of the parts after heat treatment. In this work, a high-speed quenching was used to achieve compressive residual stresses on the surface of AISI/SAE 5160 steel samples by producing high thermal gradients and interrupting the cooling in order to generate a case-core microstructure. A special laboratory equipment was designed and built, which uses water as the quenching media in a high-speed water chamber. The severity of the cooling was characterized with embedded thermocouples to obtain the cooling curves at different depths from the surface. Samples were cooled for various times to produce different hardened case depths. The microstructure of specimens was observed with a scanning electron microscope (SEM). X-ray diffraction (XRD) was used to estimate the magnitude of residual stresses on the surface of the specimens. Compressive residual stresses at the surface and sub-surface of about −700 MPa were obtained.

## 1. Introduction

Medium carbon alloyed steels are used in the manufacture of springs, which are typically hardened by oil quenching and tempering, and subsequently subjected to a warm shot peening process [1,2]. The quenching process generates tensile residual stresses on the surface and the shot peening is carried out to induce compressive residual stresses through plastic deformation [3]. The residual stresses influence the net stress applied in service, while tensile residual stresses increase the net stress range during fatigue conditions; the presence of compressive residual stresses reduce the stress range. Therefore, components with compressive residual stresses have shown considerably better performance under cyclic loading [4,5].

Aronov et al. [6] demonstrated that the use of conventional water at high agitation can reduce the film boiling stage during quenching that causes cracking and distortion. Additionally, Kobasko et al. [7] showed that with the use of highly-agitated water it is possible to generate compressive residual stresses on the surface of steel components if the quenching is interrupted before the sub-surface layers reach the martensite transformation.

Further studies have revealed that severe cooling of steel parts produced compressive residual stresses at the surface when the quenching is properly interrupted at a specific time for a particular quenching system [8,9]. The temperature difference between the surface and the core needs to be high enough that a martensite case is produced at the surface, while martensitic transformation is avoided at the center. The component experiences thermal contraction due to the cooling before the martensite transformation temperature is reached; at the surface, an expansion caused by the austenite to martensite takes place while sub-surface layers are still hot. The volumetric expansion of the surface case generates compressive residual stresses, which continue increasing up to the maximum value possible. Then, as the core starts to transform, the compressive residual stresses at the surface decrease in magnitude and eventually switch to tensile stresses due to the bulk’s expansion [10].

In the present study, an interrupted high-speed quenching (or intensive-quenching) followed by isotherm heat treatment at the Martensite start temperature (M_s_) was applied to 5160 steel samples with the goal of generating a tempered martensite case with a bainite core. This heat treatment was carried out with the scope of generating compressive residual stresses at the surface.

## 2. Materials and Methods

### 2.1. Materials

Square bars of 5160 steel were received in the hot rolled condition. The chemical composition was obtained using an optical emission spectrometer, see Table 1. 

The samples used for the quenching process consisted in square bars with a cross section of 20 × 20 mm and 100 mm in length. In order to measure the cooling curve and to analyze the severity of the cooling at different depths, 3 thermocouples were used. Blind holes of Ø 1 mm were made with electrical discharge machining (EDM) up to a middle length at 1/2 and 1/4 of the thickness and 1 mm below the surface. The holes were pre-filled with graphite and K-Type thermocouples were placed in. The graphite power ensured the tightening and good contact between the thermocouple and the sample. A ceramic sealant was employed to prevent water entering the holes during quenching. Only 2D heat transfer takes place at the center since the ratio of the thickness/length is greater than 4, thus the area of interest is at the center of the sample and quenched-ends were discarded. 

### 2.2. Quenching System

**Figure 1:**
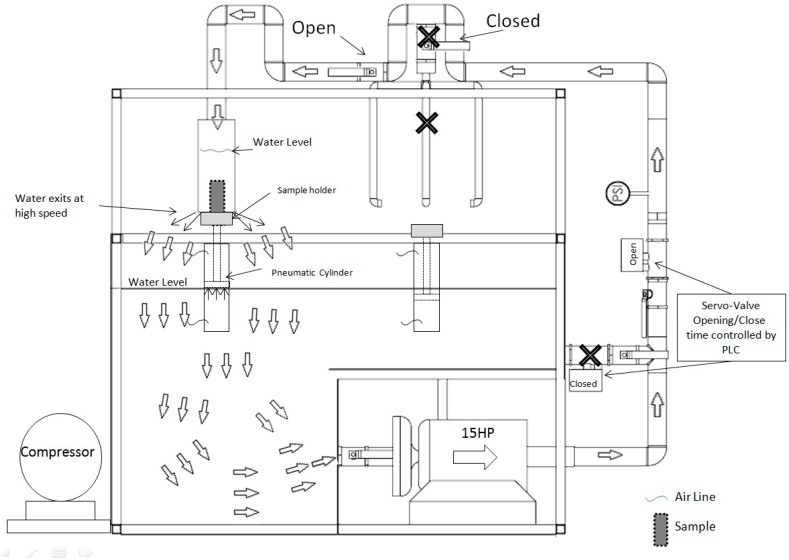
High-speed quenching system.

**Figure 2:**
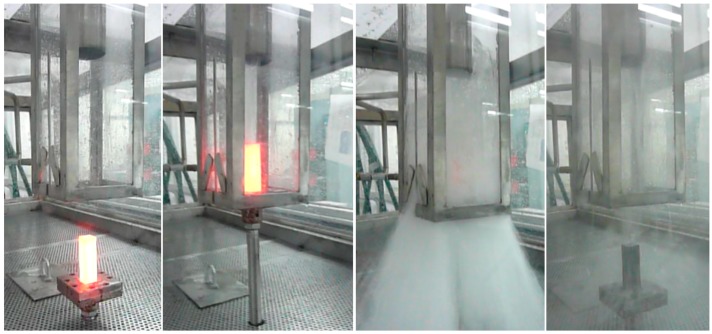
High-speed quenching chamber in operation.

A schematic representation of the quenching equipment built for this research is shown in Figure 1. The system consists of a 15 HP (horse power) centrifugal water pump, 500 liters reservoir, a Ø3 in. piping line, 2 servo hydraulic valves, air compressor, air line, 2 pneumatic cylinders, manual stopcocks, a manometer gauge, a temperature sensor, and a programmable logic controller (PLC). The water is initially recirculating into the tank with one servo hydraulic valve open and the valve of the chamber is closed until the quenching is manually started. Then, the valves switch and the water flow is redirected to the high-speed chamber during the programmed time. The system operates with a flow rate of 1325 lpm and a pressure of 4 bars. The PLC controls the dwell time for the opening and closing of the valves and the pneumatic cylinders. A sample holder is fixed in the cylinder and is operated with a button. The hot sample is placed on the holder and the start button is pressed. The cylinder is raised into the quenching chamber, and at the same time the valves switch the flow to the chamber. The cylinder takes less than one second to reach the upper position and the water flow requires 1.5 seconds to reach the outlet. In the upper position, the sample holder partially blocks the water flow exit, thus the chamber is filled and the sample remains fully immersed while the water passes at high-speed through the chamber. After the programed cooling-time is reached, the servo hydraulic valves switch again and the cylinder lowers. The PLC can control the valves with a precision of 0.1 seconds. Figure 2 shows the high-speed chamber operation.

### 2.3. Heat Treatments

**Figure 3:**
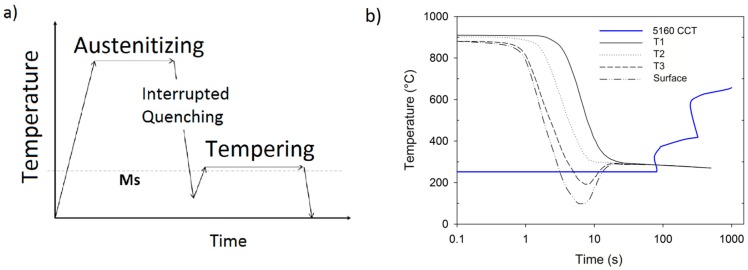
Schematic of the heat treatment cycle showing the interruption of quenching (**a**), and cooling curves at different locations (**b**).

Samples of 5160 steel were pre-heated below A_c1_ at 650 °C prior to be austenitized at 850 °C for 30 min and then water quenched in the high-speed system. The experimental matrix included different quenching times in order to interrupt the transformation at certain depths and to obtain a case-core (also called shell-core) type microstructure, which is suitable to produce compressive residual stresses. Quenching was interrupted after 2, 3, 4, and 5 seconds and different case depths were obtained. Samples were placed in a furnace immediately after quenching for 1 hour at 250 °C, which corresponds to the M_s_ temperature. Since the cooling was interrupted, the second cycle was carried out to promote the bainite isotherm in the center and at the same time to temper the martensite on the surface of the specimen. Figure 3a shows the heat treatment cycle followed. The surface temperature experiences recalescence when the cooling was interrupted and the sample was placed at 250 °C, while the cooling path of the core enters directly into the bainite zone in the CCT diagram, see Figure 3b. 

### 2.4. Characterization of Samples

One sample of each condition was characterized after heat treatment by means of scanning electron microscopy (SEM, in a JEOL JSM-6510LV Tokyo, Japan) and X-ray diffraction (XRD). Two different X-ray diffraction equipment were used; a Bruker D8 Advance (Billerica, MA, USA) equipment with copper (Cu) radiation and the powder method was used for the determination of phases of the samples; a X’pert model of the Philips (Eindhoven, The Netherlands) brand with chromium (Cr) radiation was used for the XRD evaluation of superficial residual stresses.

The XRD measurement was performed on the surface of the quenched samples and the preparation prior to the test consisted of removing the remaining oxide in the sample. For the samples evaluated by the semi-destructive technique (residual stresses with respect to the sample depth), electropolishing was employed between each step. In both cases, the crystal lattice was initially measured and analyzed using the standard Sen^2^ψ method against interplanar spacing, *d* [11]. Once the network spacing measurement was obtained, a modulus of elasticity of 205 GPa and a Poisson ratio of 0.29 were used to determine the stresses in the material using the biaxial stress method.

The non-destructive technique was used in the samples to evaluate the surface and a semi-destructive technique was carried out to assess the profile of residual stresses with respect to the depth of the sample [12]. 

**Figure 4:**
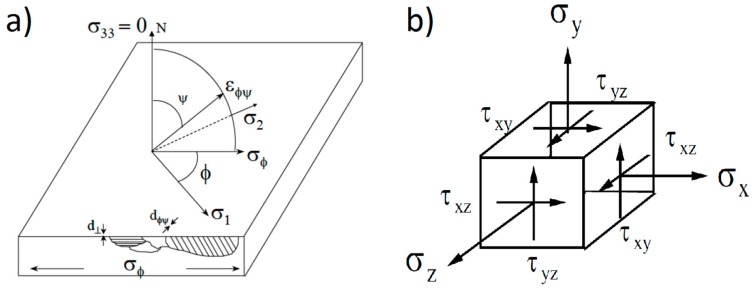
Angle and directions of stresses and unit strains in a flat sample (**a**), and stress tensor with the principal stresses schema (**b**) [13].

Figure 4 shows a schematic representation of the angles, directions of stresses, and unit strains used in the uniaxial analysis and the stress tensor with the principal stresses in three dimensions.

## 3. Results and Discussion 

### 3.1. Thermal Analysis

**Figure 5:**
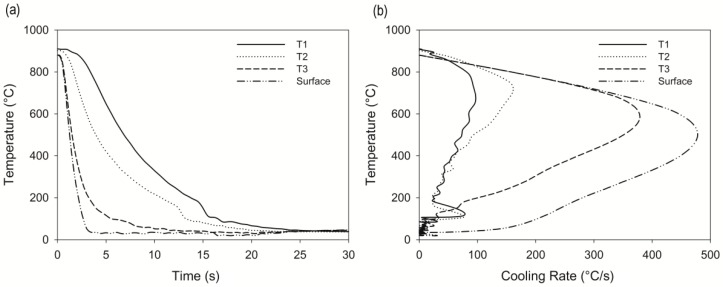
Cooling curves (**a**) and cooling rate curves (**b**); T1-½ thickness, T2-¼ thickness, and T3-1 mm below the surface.

Figure 5a shows the cooling curves and cooling curve rates obtained with embedded thermocouples in the high-speed chamber from the austenitizing temperature to room temperature. The surface temperature was calculated from the inner temperatures and use of the 2D heat transfer problem [14]. The temperature of the sample homogenized at room temperature after 25 seconds, while the surface cooled down in only 3 seconds. The highest thermal gradient observed between the surface and core was 778 °C after 3 seconds.

The cooling rate calculated for the system, shown in Figure 5b, referred to as the maximum cooling rate at the surface, was 497 °C/s. According to Kobasko [15], cooling rates above 470 °C/s result in a reduction of the cracking probability, and due to the severity of cooling, the process is referred to by some authors as intensive quenching. 

In order to produce compressive stresses, the quenching must be stopped when the thermal gradients are high enough that martensite and austenite are present within the sample. From the cooling curves of Figure 5, it can be noted that the thermal gradients between the surface and the core are maximum in the first 6 seconds. Thus, the cooling times selected were 1, 2, 3, 4, 5, and 6 seconds, followed by immediate isotherm in a furnace at 250 °C.

**Figure 6:**
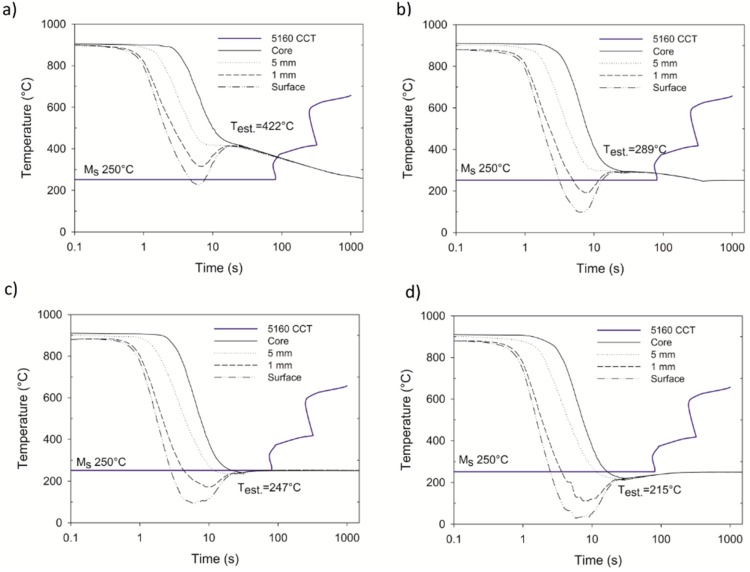
Cooling curves obtained in the high-speed chamber for (**a**) 2, (**b**) 3, (**c**) 4, and (**d**) 5 s.

The quenching system was programmed with 1 second steps starting with one second up to 6 seconds. For each cooling time, the cooling curve was obtained to evaluate the temperature distribution and the stabilization temperature of the sample after the cooling was interrupted. Figure 6 shows the cooling curve for quenching times of 2, 3, 4, and 5 seconds. After one second of quenching, the surface temperature did not reach the M_s_ temperature, therefore, no transformation occurred. 

From the thermal data displayed in Figure 6, it can be expected that the optimal conditions to achieve the case-core microstructure are between 3 s and 4 s, since the 2 s condition was not enough time to cool the surface below the M_s_ temperature and to promote the martensite transformation; while in the 5 s condition, the temperature of the core dropped below the M_s_, which would be expected to reduce the compressive stresses due to the transformation expansion of the core. In the conditions corresponding to 3 and 4 s, a sufficient thermal gradient was observed between the surface and the core, such that martensite and austenite are present at the same time; thus, compressive residual stresses on the surface of the sample are expected to some extent since the hardened case experiences expansion when the martensite transformation takes place, while the core that remains as austenite experiences thermal shrinkage. By observing the thermocouple at the core location for the different conditions, it can be observed that 4 s is the maximum time before the core reaches the transformation temperature. The thermal path of the conditions of 2 and 3 s at the core enters directly into the bainite nose.

**Figure 7:**
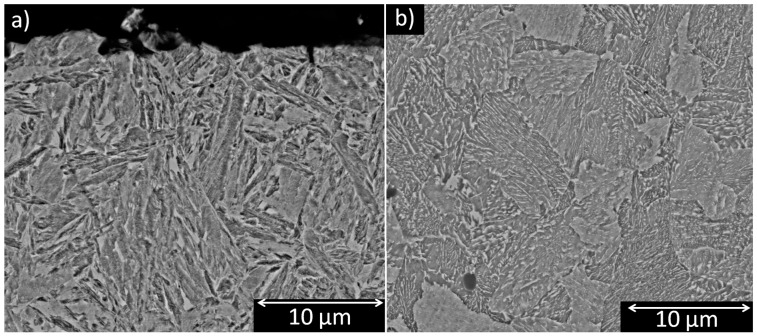
Metallography observed by SEM of 3 s showing tempered martensite at the surface (**a**) and columnar and nodular bainite at the core (**b**).

The sample of the 3 s condition stabilized at 289 °C and showed a bainitic microstructure in the core due to a high thermal gradient during the quenching treatment creating favorable conditions for the formation of a core-shell microstructure, as depicted in Figure 7. Canale [16] described an increase of compressive residual stress due to the expansion of martensite on the surface and contraction of the core.

### 3.2. X-ray Diffraction

**Figure 8:**
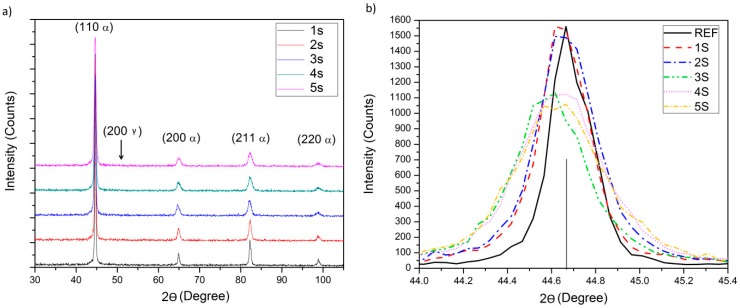
X-ray diffraction pattern (**a**) and close-up of the principal peak (110-α) (**b**).

Firstly, x-ray diffraction was performed to evaluate the presence or absence of retained austenite. Figure 8a displays the X-ray diffraction pattern corresponding to cooling times of 1, 2, 3, 4, and 5 s, followed by the isotherm treatment at the M_s_ temperature (250 °C). 

The bcc of retained austenite peak corresponding to the plane (2 0 0 γ) was not observed in any condition, thus it can be stated that the tempering caused the transformation of the untransformed austenite to bainite [17]. Upon evaluation of angle 2θ, the samples corresponding to 2, 3, 4, and 5 s showed affinity with bainite indexed cards. 

The presence of residual stresses is reflected in the width of the x-ray diffraction peak and consequently in its intensity. A detailed analysis of the principal peak (1 1 0 α) is displayed in Figure 8b. The peak widens and a reduction is observed in the intensity axis. The peak suffered a width increase as the cooling time increased when compared with a reference of the same material without heat treatment; that is, with no residual stresses (solid line). 

The diffraction peak width at 2w or FWHM (full-width at half-maximum), which is related to the hardening [18,19], is displayed in Table 2 along with the angle, 2θ’, and *d_2*θ’*_*. The sample of 5 s exhibited the greater width increment with a 2w value of 0.49, followed by conditions 4, 3, and 2 in order of peak deformation. Using the Bragg’s Law, the interplanar d-spacing can be calculated using the stress free distance as a reference to obtain the crystallographic strain. The stress free d-spacing considered was 1.1705 (Å).

#### 3.2.1. Residual Stresses

**Figure 9:**
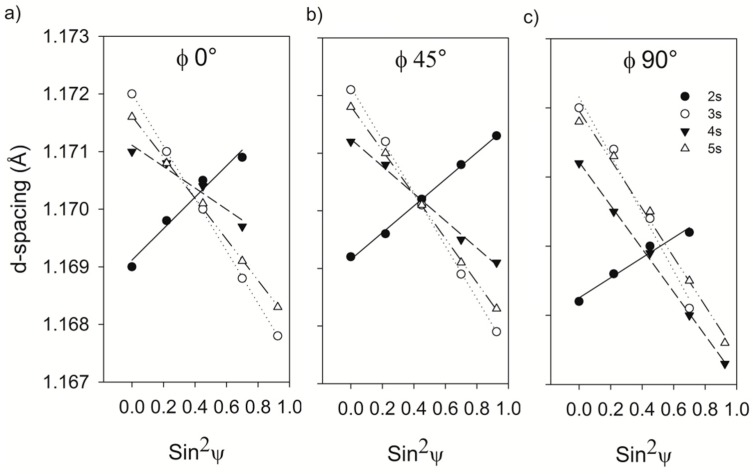
Analysis of d_φψ_ vs sin^2^ ψ.

Additionally, from the analysis of d_φψ_ vs sin^2^ ψ, the sign of the residual stresses can be determined. A negative slope means the presence of tensile stresses while a positive slope corresponds to compressive stresses [12]. Figure 9 shows the d_φψ_ vs sin2 ψ pattern obtained from X’pert Data Collector software for tilt angles of 0°, 45°, and 90°. It can be observed that the 2 s condition slope is positive, which means that it is under tensile residual stresses, and the conditions of 3, 4, and 5 s are under compressive ones. Table 3 shows the uniaxial stress analysis of the different conditions.

A biaxial analysis was calculated from the stress tensor to obtain the principal stresses, see Table 4. The 3 s condition was the condition that showed the maximum magnitude of compressive stresses with a value of –628 MPa in the principal stress.

#### 3.2.2. Residual Stress Profile

**Figure 10:**
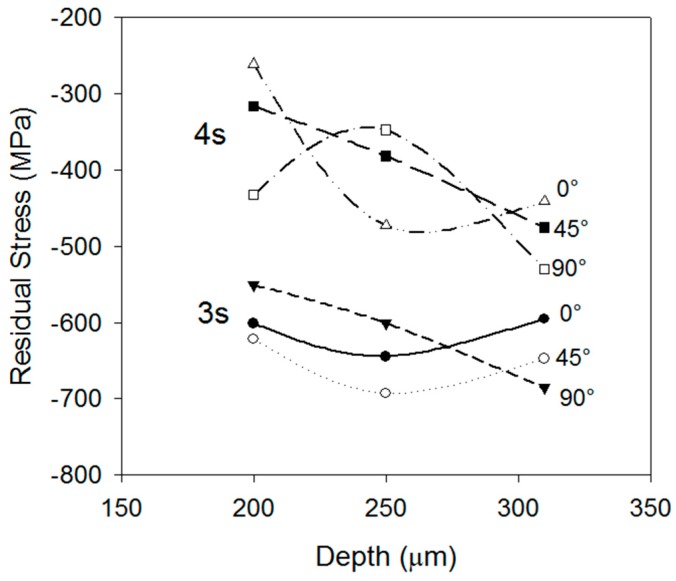
Residual stress profile of the conditions of 3 s and 4 s.

The residual stress profiles as a function of the depth were obtained by performing x-ray measurements on the surface and then a surface layer was dissolved by means of an electropolishing process. The residual stresses were measured at different depths down to a 0.31 mm depth. Figure 10 displays the residual stress profiles for 3 and 4 s, showing a minimum value of −698 MPa at a 0.26 mm depth and −490 MPa at a 0.26 mm depth, respectively. From the residual stress evaluation and the cooling curves, it can be concluded that the reduction in magnitude of the compressive stresses from the 3 s to the 4 s condition produces very high volumetric expansion caused by the martensite transformation of deeper layers.

## 4. Concluding Remarks

The quenching severity obtained in high-speed quenching is capable of producing very high thermal gradients at the beginning of cooling greater than 750 °C between the surface and the core, making it possible to produce a shell-core microstructure. 

The shallow hardening induced compressive residual stresses at the surface. From the slope of the d-spacing vs Sin^2^ψ, it was concluded that the conditions of 3, 4, and 5 s resulted in compressive residual stresses and this was corroborated with the uniaxial and biaxial stress analysis. 

The maximum magnitude of the compressive residual stresses was found to take place with 3 s of cooling. The residual stresses at the surface were −621MPa and had a maximum value of −700 MPa at 250 μm below the surface. The shell-core microstructure consisted in tempered martensite at the surface and bainite at the core. 

The interruption time is critical in the retention of compressive residual stresses, since further cooling of inner layers will reduce compressive stresses when the subsurface layers expand due to martensite transformation. 

## Figures and Tables

**Table 1:** Chemical composition of the studied steel.

Element	C	Mn	P	S	Si	Ni	Cr	Cu	Nb	Ti	Al
**wt%**	0.58	0.885	0.017	0.016	0.26	0.01	0.78	0.013	0.003	0.0038	0.0264

**Table 2:** Diffraction peak width at maximum height (FWHM parameter).

Sample	Intensity	2w	2θ’	*d_2*θ*’_*
1 s	1562	0.25	44.665	2.0274
2 s	1497	0.35	44.655	2.0276
3 s	1134	0.39	44.585	2.0308
4 s	1123	0.45	44.615	2.0295
5 s	1058	0.49	44.635	2.0287

**Table 3:** Uniaxial stress analysis.

Angle	2s		3s		4s		5s	
φ (°)	σ_φ_ (MPa)	τ_φ_ (MPa)	σ_φ_ (MPa)	τ_φ_ (MPa)	σ_φ_ (MPa)	τ_φ_ (MPa)	σ_φ_ (Mpa)	τ_φ_ (MPa)
0	358.3	6.6	-601.1	20.1	–261.2	9.9	–490.7	22.9
45	278	19.9	-621.6	–5.6	–316.3	19.4	–508.5	19.9
90	189.7	12.6	–551	–9.3	–431.8	17.4	–470.6	31

**Table 4:** Biaxial analysis showing the stress tensor and principal stresses.

Sample	Stress Tensor (MPa)				Principal Stresses (MPa)			
		358.3	4	6.6		358.4	0	0
2s	σ_ij_=	4	189.7	12.6	σ_ij_=	0	189.6	0
		6.6	12.6	0		0	0	0
		−601.1	−45.5	20.1		−628	0	0
3s	σ_ij_=	−45.5	−551	−9.3	σ_ij_=	0	−524.1	0
		20.1	−9.3	0		0	0	0
		−261.2	30.2	9.9		–256	0	0
4s	σ_ij_=	30.2	−431.8	17.4	σ_ij_=	0	−437	0
		9.9	17.4	0		0	0	0
		−490.7	−27.9	22.9		−510.3	0	0
5s	σ_ij_=	−27.9	−470.6	31	σ_ij_=	0	−451	0
		22.9	31	0		0	0	0
